# Aramid-Zirconia Nanocomposite Coating With Excellent Corrosion Protection of Stainless Steel in Saline Media

**DOI:** 10.3389/fchem.2020.00391

**Published:** 2020-05-19

**Authors:** Ahmed Abdel Nazeer, Fakhreia Al Sagheer, Ali Bumajdad

**Affiliations:** ^1^Chemistry Department, Faculty of Science, Kuwait City, Kuwait; ^2^Electrochemistry Laboratory, Physical Chemistry Department, National Research Centre, Giza, Egypt

**Keywords:** 316L stainless steel, corrosion protection, hydrophobic nanocomposite coating, surface properties, aramid-ZrO_2_ film

## Abstract

Resistance to stainless steel corrosion in marine-based industries requires more research into materials with an improved surface and enhanced protection by utilizing surface coatings. Herein, a thermally stable aramid–zirconia nanocomposite has been successfully prepared using the sol–gel method to produce a zirconia network-structure bonded to the polymer chain. Using thermal gravimetric analysis (TGA), the residue mass of zirconia retained after the thermal degradation of aramid-zirconia film was determined and found to be 10% by mass. The investigated nanocomposite (using 10% zirconia) was coated on the stainless-steel surface through a facile and effective spin coating method and its protection was examined in saline solution (3.5% NaCl). The aramid–zirconia nanocomposite coating (Ar-Zr10) was found to provide an outstanding corrosion resistance to steel surfaces which led to protecting it against the corrosive marine environment. The electrochemical impedance (EIS) measurements were carried out to evaluate steel resistance against dissolution in chloride solution in the absence and presence of the investigated coatings showed a corrosion protection efficiency of 99.3% using Ar-Zr10 compared to 92.1% using pure aramid. Moreover, the potentiodynamic polarization (PDP) plots showed a pronounced decrease in the corrosion current values which confirmed the formation of a passive layer which mitigated the corrosion reaction and ions diffusion. The water contact angle of stainless-steel coated with pure aramid and the aramid–zirconia was found to be 84.2° and 125°, respectively, confirming the hydrophobic nature of the hybrid coating Ar-Zr10. On the other hand, the results achieved through the electrochemical and surface techniques were used to clarify the protection mechanism. The aramid–zirconia nanocomposite coating showed a remarkable protection performance by controlling the charge transfer at the interface between the steel alloy and the electrolyte which prevented the alloy dissolution.

## Introduction

Protection of metals and alloys against corrosion is of great importance in the modern metallic finishing industries due to different economic and environmental considerations. According to literature, annual costs for corrosion are now approximately $1 trillion, or 6 per cent of the United States domestic GDP (Koch et al., 2005). Moreover, corrosion problems, especially leakage in pipelines, tanks, tubes, and other equipment, arise from the different operating problems and lack of maintenance resulting in a lot of economic problems. Stainless steels are pronouncedly used in the industrial fields because of their corrosion resistance, accessibility, low cost, great mechanical characteristics, and ease of fabrication (Yaya et al., 2011).

The development of a passive chromium oxide film over the stainless-steel surface is mainly responsible for its corrosion resistance (Caselis et al., 2012). Nevertheless, stainless steels are prone to various corrosion forms including pitting, inter-granular, and crevice corrosion in aggressive chloride media (Shahryari et al., 2009).

Many efforts have been spent on the research of corrosion with different strategies to suppress the different corrosion types. In order to improve the alloys lifetime, the corrosion processes must be controlled by an appropriate design and choice of their metal composition and by the effective coatings or inhibitors.

Polymeric coatings were commonly used as a block layer among the corrosive ions and the metallic surface in order to prevent corrosion (Gebhardt et al., 2012). For reducing or preventing the metals and alloys corrosion rate, particularly when cost-efficacy is regarded, the use of hydrophobic nanocomposite coatings is important.

Hybrid nanocomposites using the carbonaceous compounds to work in coatings are promising alternatives to zinc and chromate coatings (Nazeer and Madkour, 2018). Polymer coatings have demonstrated metallic corrosion protection but badly adhere on their surfaces. Moreover, the nanostructured films are promising, but have an intrinsic porosity which generates water and ion channels to penetrate the film and corrode the substrate (Mohamed et al., 2015). The development of hybrid nanocomposites composed of several parts is becoming more important to effectively provide corrosion suppression and adhesion to the metallic substrates (Radwan et al., 2018).

Nanocomposites are multiphase solid materials composed of two or more different materials with at least one element with a size <100 nanometers. Polymeric nanocomposite coatings have attracted researchers interest due to their valuable and promising applications in different fields due to their unique physicochemical properties compared with using each component individually (Wang et al., 2013; Neella et al., 2017). Using conducting polymers in fabricating superhydrophobic coatings will offer a more pronounced corrosion protection for steel alloys by shifting the corrosion-potential in the anodic direction, which will result in the passivation of the substrates (Tallman et al., 2001). There are a lot of works dealing with using the conducting polymers as anticorrosion coating for steel alloys such as polypyrrole, poly(vinylcarbazole), and polyaniline (Frau et al., 2010).

Chang et al. (2012) reported using a nanocomposite of PANI/graphene for the corrosion protection of steel. Also, polythiophene has been used as a superhydrophobic conducting polymer for the protection of steel substrates from corrosion attack effectively. The protection efficiency of this polymer is attributed to its prevention of water absorption onto the coating and hence the prevention of the diffusion of the corrosion products through the coating and suppression of the metal dissolution (de Leon et al., 2012). In the literature, there are few studies related to the use of inorganic-organic composites in coatings. In this case, nanoparticles (inorganic) are one component of the composite and polymer (organic) is the second component. The nanoparticles introduce a high surface-to-volume ratio which changes the composite properties if compared with the bulky component materials. Also, introducing nanoparticles to the polymer are known to improve the electrical and/or thermal conductivity, mechanical strength, and/or toughness of the resulting composite. For instance, using nanocomposites from a nanometer-sized ZnO and TiO_2_ with polyaniline showed pronounced improvements in corrosion protection compared with the polyaniline alone (Radhakrishnan et al., 2009).

ZrO_2_ nanoparticles have an efficient antibacterial effect with excellent anti-corrosion properties, high strength, strong wear, and thermal resistance, good hardness, and remarked chemical resistance (Nabi et al., 2011; Jangra et al., 2012). These outstanding properties provide a strong opportunity for industrial application, as ZrO_2_ NPs are environmentally acceptable due to their non-toxicity (Cho and Ko, 2013). Various studies have reported on the application of ZrO_2_ nanostructured ceramic coatings to protect different metals and alloys, such as ZrO_2_ (Liu et al., 2002), ZrO_2_-TiO_2_ (Garg et al., 2015), Al_2_O_3_ – yttria stabilized ZrO_2_ (Amaya et al., 2009), and SiO_2_-TiO_2_ -ZrO_2_ (Bautista-Ruiz et al., 2014) which showed improved corrosion-resistance in a corrosive media. Interestingly, the incorporation of zirconium oxide nanoparticles into the polymeric structures demonstrates remarkable physical and chemical properties and improves the heat resistance of composites which make it difficult to crack (Masim et al., 2019). Ramanathan et al., studied the protection of mild steel using a composite of the epoxy resins with the nano-zirconia coating and the results showed good adhesion to the metallic surface with excellent corrosion resistance (Ramanathan and Balasubramanian, 2016). Also, zirconia-epoxy nanocomposites have been investigated for the mild steel protection in 3.5% NaCl solution and the results showed an improved corrosion performance (Behzadnasab et al., 2011). In another report, hybrid coatings based on graphene oxide–zirconia dioxide/epoxy were fabricated and their corrosion protection for steel was investigated and effectively prevented the steel substrate from corrosion attack (Haihui et al., 2016).

Due to their pronounced mechanical-strength and efficient thermal-stability, polyimides (PI) and aramids (Ar) are increasingly being used to prepare high-performance nanocomposites (Abadie and Rusanov, 2007; García et al., 2010). Aramids (Fu et al., 2010) e.g., Kevlar® and Nomax®, provide remarkable mechanical properties with stiffness and strength, thus making these the perfect candidates for the development of new composites.

This work proposes to synthesize a pure aramid and aramid-zirconia hybrid film using a modified procedure of the method mentioned elsewhere (Ahmad et al., 2007). This modification was meant to warrant the matrix of polymers by promoting strong chemical bonding within the organic polymer components. The synthesized hybrid organic-inorganic film was examined as an effective coating for the protection of a stainless-steel alloy in 3.5% NaCl (typical concentration in marine environment). The protection efficiency of the designated coatings against corrosion was explored via EIS and PDP techniques complemented with scanning electron microscopy (SEM).

## Experimental Procedure

### Material

Monomers; 1,3 phenylene diamine, 1,4 phenylene diamine, and terephthaloyl chloride (TPC) were of analytical grades with a purity of 99% and a product of Sigma Aldrich. The 99.8% anhydrous dimethylacetamide (DMAC) solvent is a product of Sigma Aldrich. Zirconium (IV) propoxide solution (70 wt.% in 1-propanol) (Mw: 327.57) was also obtained from Sigma Aldrich. The other chemicals and reagents were of AR grades and were used as obtained.

The electrochemical studies were performed on AISI 316LSS samples with thickness (5 mm) and 2 cm^2^ surface area. AISI 316LSS composition (in wt.%) is as follows: Cr(18%), C(0.08%), Ni(14%), Mn(2.0%), Si(0.1%), P(0.45%), S(0.03%), N(0.1%), Mo(3%), and the rest iron. The corrosive media used is (3.5% NaCl) with pH (6.5–7.0) which matches with sea water from Arabian Gulf. The 316L SS specimens were finished with SiC paper (400 to 1,200 μm) grit followed by polishing using a 3, 9-micron diamond-paste and with Al_2_O_3_ solution and finally ultrasonicated with acetone, ethanol, and bidistilled water for 10 min.

### Preparation of Aramid-Zirconia Hybrid

The linear aramid chain synthesis was performed through the polymerization of 1, 4-phenylene diamine (and/or 1,3-phenylenediamine) and TPC at a low temperature. It was evident that, as the molecular weight increases, the polymer appears insoluble in the organic solvent. The solubility of the resin in DMAC was improved by using an optimal number of kinks in the linear amide chain. A mixture of 1,4- and 1,3-phenylenediamine, (0.050 mol) in molar ratio 35:65, respectively was inserted in a conical flask (250 ml). Then, 150 g of solvent DMAC was introduced and stirred for 30 min till the mixing was complete. 0.050 mol of TPC was added under complete anhydrous conditions. The mixture was continuously stirred for another 24 h after which the polymerization reaction was supposed to be complete. Then, the calculated amount of zirconium (IV) propoxide in DMAC was added to the aramid solution and stirred for 4 h. To start the Sol-Gel reaction, a stoichiometric quantity of water in DMAC was added to the solution. HCl created during polymerization acts as a catalyst. The solution was then stirred at 60°C for 12 h to finalize hydrolysis and the condensation of the zirconia inorganic network. Finally, the pure aramid and the Ar-Zr10 composite were coated on the steel surface as we will mention in the coating step. It is worth mentioning here that the amount of ZrOx loading (10%) was selected based on our experience that 20% often results in particle agglomeration and 5% gives low inorganic yield in the polymer matrix (this will be reflected on the film tensile strength, and hence, on the corrosion protection capability).

### Coating Films Fabrication

The coating was prepared by drop casting the pure aramid or the aramid-zirconia composite onto the studied AISI 316L surface and drying for 3 min at 100°C. Then, the substrates were treated thermally at 200°C and then immediately cooled to room temperature. Additionally, the control specimen of bare AISI 316L alloy was used without any coating.

### Characterization

Characterization of the prepared Aramid-zirconia nanocomposite film was performed using TGA, FTIR, XRD, XPS, and drop shape analysis system (Kruss DSA10 MK2). TGA curves were attained after heating the tested sample (10°C/min.) up to 800°C in a dynamic atmosphere of synthetic air, via TA-50 Shimadzu thermal system (Japan). FTIR spectra were recorded at 4000-400 cm^−1^, using a KBr-supported thin disc of the nanocomposite film via 2000 Perkin-Elmer FT-IR spectrometer (USA). XRD analysis was performed using a D8 Advance diffractometer with a Cu target and nickel filter with CuKα radiation of λ = 0.154056 nm. XPS measurements were carried out using a Thermo ESCA Lab 250xi with Mg Kα radiation (1253 eV).

### Electrochemical Measurements

For electrochemical measurements, 1 cm^2^ Pt-sheet and saturated calomel electrode (SCE) were used as counter and reference electrodes, respectively, while AISI 316L (1.0 cm^2^) was used as a working electrode (WE). A potentiostat model Gamry 3000 was used for electrochemical experiments. PDP measurements were recorded after 20 min to reach equilibrium at a scan rate of 1 mV/s. EIS tests were conducted in the frequency range (10^5^–0.1 Hz).

## Results and Discussion

### Characterization of Pure Aramid and Ar-Zr10 Composite

**Figure 1:**
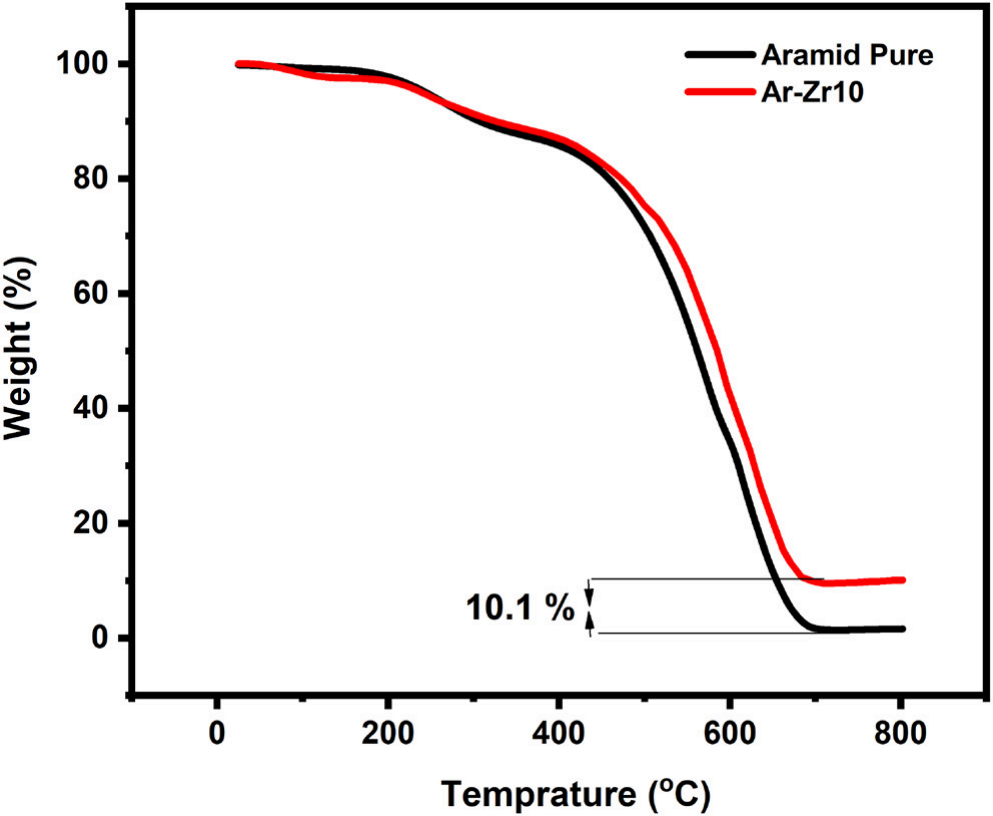
Thermogravimetric curve for the pure Aramid and Aramid-zirconia hybrid film (10%). The heating rate was 10°C/min in a dynamic atmosphere of air showing the zirconia content.

TGA analyses were performed for the pure Aramid and the Aramid-zirconia hybrid films (10%) under synthetic air in the temperature range (25–800°C), as shown in Figure 1. The thermal decomposition for the aramid-zirconia hybrid film (10%) was monitored through the full temperature range showing two main decomposition steps; the first one maximized at about 265°C, corresponding to the water from some of the unhydrolyzed zirconia network. The second step of the thermal decomposition of the material was generally in the range of 500–600°C, which maximized at about 526.1°C related to the decomposition of the byproduct of the aramid main chain.

The residue mass retained following the degradation process at 800°C is 10.1% for Aramid-zirconia hybrid film, which is in accordance with the zirconia content in the hybrid film (10% by mass) and this evidence confirms the validity of the sol-gel preparation method. For the thermal decomposition of the pure aramid film, it is clear that the films undergo 100% mass loss after the thermal degradation up to 800°C.

**Figure 2:**
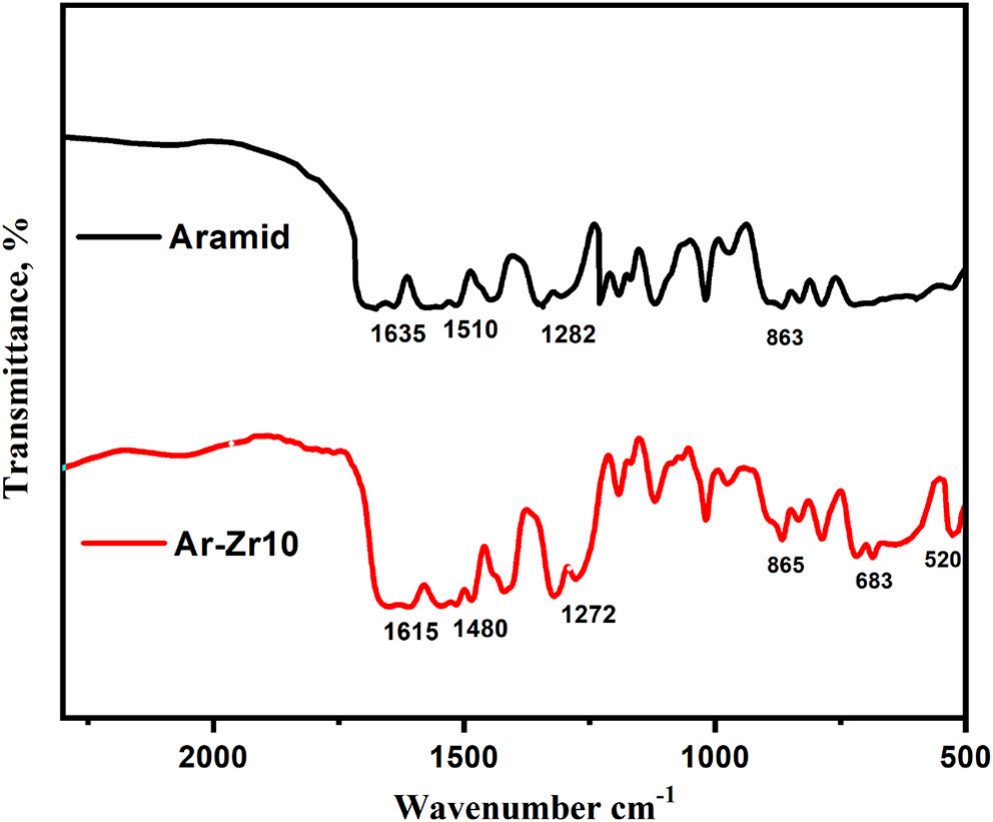
FT-IR spectrum recorded for the pure aramid and Ar-Zr10 composite.

FTIR analysis was performed on pure aramid film and Ar-Zr10 composite as shown in Figure 2. The spectra monitor used an absorption band at 1,635 cm^−1^, due to υC=O and υC=N stretching which is a characteristic peak of Amide I (Ahmad et al., 2007). Amide II mode is monitored with the band observed at 1,480 cm^−1^, which corresponds to interacting υC=N and δC-N-H vibrations. Also, the absorption band at 1,272 cm^−1^ due to δ(NH) and δ-O-C-N vibrations is characteristic to the Amide III (secondary amide) (Ahmad et al., 2007). The relatively lower intensity of the amide II mode signifies the presence of hydrogen bonding. The peak at 520 cm^−1^ in the case of the Ar-Zr10 composite allocated to the Zr-O confirms the reaction of polymer and precursor (Liu et al., 2014).

**Figure 3:**
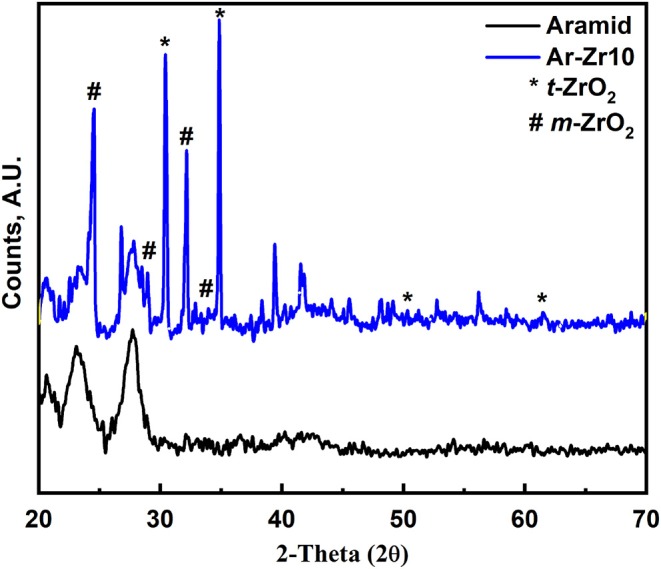
XRD pattern for the pure aramid and Ar-Zr10 composite.

XRD patterns for pure aramid and Ar-Zr10 composite film were recorded in Figure 3. The diffraction peaks of the pure aramid showed a crystalline nature with main diffraction peaks at 2θ = 20.6°, 22.7°, 28.1° corresponding to 110, 200, and 004 planes, respectively (Ifuku et al., 2014). The pattern for Ar-Zr10 composite showed the appearance of new additional peaks related to ZrO_2_. The pattern revealed a mixed crystal structure of tetragonal and monoclinic ZrO_2_. The tetragonal phase of ZrO_2_ contained XRD peaks at 2θ = 30.2°, 35.2°, 50.6° and 60.2° (Figure 3) which are identical to those in JCPDS No. 80-0965 (Bumajdad et al., 2018). The XRD peaks associated with monoclinic zirconia occur at 2θ = 24.2°, 28.2°, 31.4°, and 34.3° in accordance with JCPDS No. 37-1484 (Bumajdad et al., 2018). The average crystallite size (D) of the cubic phase was estimated using the Debye Scherer's equation (1).

(1) $$\begin{array}{l} \text{D}=0.89\text{λ}/\;\left(\beta \text{cos}\theta \right) \end{array}$$

where λ is the wavelength of the X-ray beam, β is the full width at half-maximum (FWHM) of the more intense peak of ZrO_2_ NPs, and θ is the angle of diffraction. The main peak in this diffractogram at 34.3° is related to monoclinic crystal structure. The average crystallite size of the monoclinic phase is 21.8 nm.

**Figure 4:**
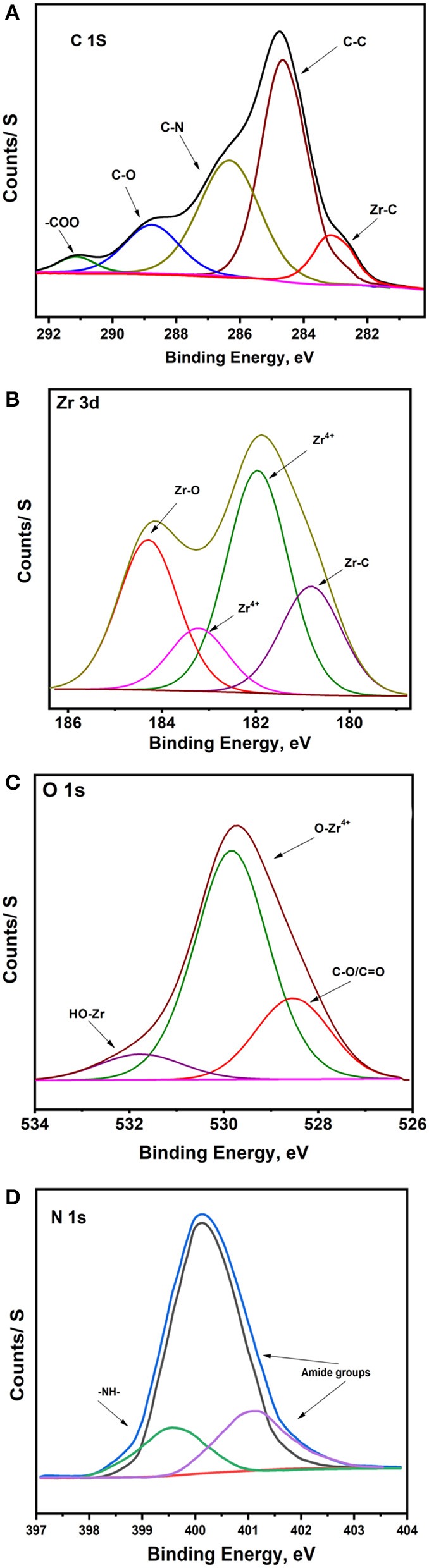
Deconvoluted XPS of **(A)** C(1s), **(B)** Zr(3d), **(C)** O(1s), and **(D)** N(1s) spectra obtained for Ar-Zr10 composite.

The XPS data of the Ar-Zr10 composite film are depicted in Figure 4 which illustrates the spectra of C(1s), Zr(3d), O(1s), and N(1s). The C(1s) spectra (Figure 4A) showed a peak at 284.6 eV attributed to C–C bonds which represent amorphous carbon or adventitious carbon (Li et al., 2019). The peak at 286.3 eV is ascribed to the C-N groups of aldehyde products while the binding energy peak at 288.3 eV is related to carbon of C-O species (Li et al., 2019). The peak at 291.1 eV is characteristic of the COO carboxyl groups. Also, the peaks positioned at around 283 eV are attributed to Zr–C bond (Liu et al., 2019).

The Zr 3d spectrum is composed of two peaks (Figure 4B) with binding energies of 180.8 and 182.0 eV, which are related to Zr 3d_5/2_ while the peaks at 183.2 and 184.4 eV are ascribed to Zr 3d_3/2_, with an energy difference of 2.4 eV. The peaks and peak separation are a consequence of the presence of Zr^4+^ ions in ZrO_2_ (Ren You et al., 1999). Moreover, Zr 3d spectra displays the presence of reduced Zr species and Zr^(4−x)+^ ions in agreement with previous reports (Teeparthi et al., 2018). Also, deconvolution of the O1s peak in the XPS spectrum of this material (Figure 4C) showed that it consists of three peaks, one at 528.5 eV which likely corresponds to C-O, C=O, or OH type oxygens in other adsorbed species (Huang et al., 2019) and two at 530.0 and 531.7 eV which are characteristic of lattice oxygen in the O^2−^ state corresponding to the metal oxide ZrO_2_ and Zr-OH, respectively (Cubillos et al., 2014; Teeparthi et al., 2018). N1s deconvolution reveals three peaks at 399.5, which is attributed to N–H, and the other peaks at 400.1 and 401.2 eV attributed to the nitrogen in the amide group (Duong et al., 2014).

### Potentiodynamic Polarization Measurements

**Figure 5:**
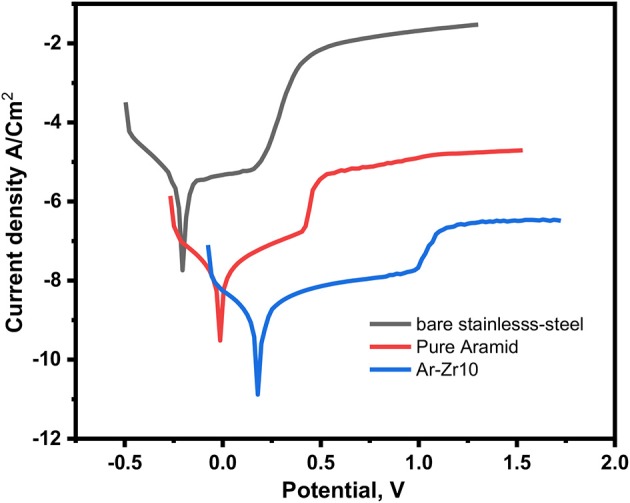
PDP curves for bare stainless-steel alloy alone and coated with aramid and Ar-Zr10 hybrid in 3.5% NaCl solution.

PDP measurements are conducted to provide an understanding of the kinetics electrochemical corrosion parameters. Figure 5 shows the Tafel polarization anodic and cathodic curves for uncoated stainless-steel alloys and coated with pure aramid and the composite of aramid-zirconia (Ar-Zr10) in 3.5% NaCl solution at room temperature. The corrosion parameters like corrosion potential (E_corr_), corrosion current density (j_corr_), polarization resistance (R_p_), and corrosion rate (P_i_) were calculated.

The R_P_ and P_i_ were measured using equations 2 and 3 (Shi et al., 2010; Argade et al., 2012):

(2) $$\begin{array}{l} {\text{R}}_{\text{p}}=\frac{\beta_{a}\beta_{c}}{2.3\left(\beta_{a}+\beta_{c}\right)j_{corr}} \end{array}$$

with β_a_ and β_c_ are the anodic and cathodic Tafel slopes, respectively.

(3) $$\begin{array}{l} {\text{P}}_{\text{i}}=22.85\;{\text{j}}_{\text{corr}} \end{array}$$

Relative to the bare stainless-steel alloy, the corrosion current of the coated alloys was decreased considerably. Using the aramid coating improved the stainless-steel corrosion resistance from 21.4 to 132.9 kΩcm^2^ as well as decreasing the current density from 2.5 × 10^−5^ to 6.3 × 10^−7^ A/cm^2^. Extra considerable decrease in the current density was recorded by aramid-zirconia (10%) composite coating, reaching 5.3 × 10^−9^ A/cm^2^. The presence of zirconia in the hybrid coating exhibited a marked corrosion resistance with respect to the bare stainless-steel alloy.

The corrosion of steel alloys and the rust formation occurs as follows (Radhakrishnan et al., 2009):

(4) $$\begin{array}{l} \text{Fe}\to \text{F}{\text{e}}^{2+}+2{\text{e}}^{-} \end{array}$$

(5) $$\begin{array}{l} \text{F}{\text{e}}^{2+}\to \text{F}{\text{e}}^{3+}+{\text{e}}^{-} \end{array}$$

(6) $$\begin{array}{l} {\text{O}}_{2}+2{\text{H}}_{2}\text{O}+4{\text{e}}^{-}\to 4\text{O}{\text{H}}^{-} \end{array}$$

(7) $$\begin{array}{l} 2\text{F}{\text{e}}^{2+}+{\text{O}}_{2\left(\text{g}\right)}+2{\text{H}}_{2}\text{O}\to 2\text{FeOOH}+2{\text{H}}^{+} \end{array}$$

So, the presence of H_2_O and O_2_ is important for the steel dissolution. Using a barrier coating is vital to control this corrosion by preventing the diffusion of these molecules and ions to the steel surface. The efficient coat that can reduces the hydrolyzed water content led to a decrease in the corrosion current of the anodic metal dissolution as well as an increase in the corrosion inhibition where the coated layer can act as a passivating layer controlling the corrosion and ions diffusion.

According to results, the higher decrease in the j_corr_ and R_p_ in case of the Ar-Zr10 composite is related to its hydrophobicity and its capability to form a stable passive layer to protect the metallic surface and prevent the corrosive ions from reaching the surface, which hinders the hydrogen evolution reaction.

Additionally, from P_i_ values, it is noticeable that the presence of the Ar-Zr10 composite (0.43 mm/year) led to a marked decrease compared to the pure aramid (27.12 mm/year) and the bare stainless-steel alloy (108.2 mm/year). These results confirm the significant improving of the protecting ability in the presence of the investigated Ar-Zr10 composite.

From the potentiodynamic polarization curves, the Tafel slopes decreased in the presence of aramid compared to the bare stainless-steel. Additional reduction was recorded using the Ar-Zr10 composite, supporting the improved protective mechanism.

Additionally, the E_corr_ in the presence of the pure aramid coating was more positive compared to the stainless steel alloy with potential value shifted to the noble direction from −0.21 (for stainless-steel) to −0.01 V/SCE (for pure aramid). Moreover, the aramid-zirconia (10%) composite coating exhibited a marked shift in the potential to a more noble direction compared to the pure aramid coating to potential 0.18 V/SCE, suggesting a delaying in the corrosion initiation, which indicates the better anti-corrosion performance of the investigated Ar-Zr10 hybrid compared to the pure aramid (Nazeer et al., 2019). It is obvious that the presence of the aramid-zirconia (10%) composite displayed a passivating behavior at a more positive potential around 0.99 V/SCE.

The presence of H_2_O and O_2_ molecules is a key factor in stainless steel alloy corrosion. It is necessary to use the coating as a barrier to prevent corrosion by suppressing the H_2_O/O_2_ molecules from the diffusion to the stainless-steel surface. In this work, the marked corrosion inhibition observed in the presence of the composite coating could be explained by the Ar-Zr10 coating acting as an efficient passive layer toward the ions diffusion, which resulted in suppressing the corrosion. This phenomenon can be related to the ZrO_2_ present in the coating, which acts as a barrier to electrons' and ions' movement among the substrate and the solution, which enhances the stainless-steel corrosion resistance even in severe chloride media.

### Electrochemical Impedance Spectroscopy

EIS spectroscopy was performed to examine the mechanism of the electrochemical reactions between the bare stainless-steel surface and the investigated coatings after exposure to 3.5% NaCl.

**Figure 6:**
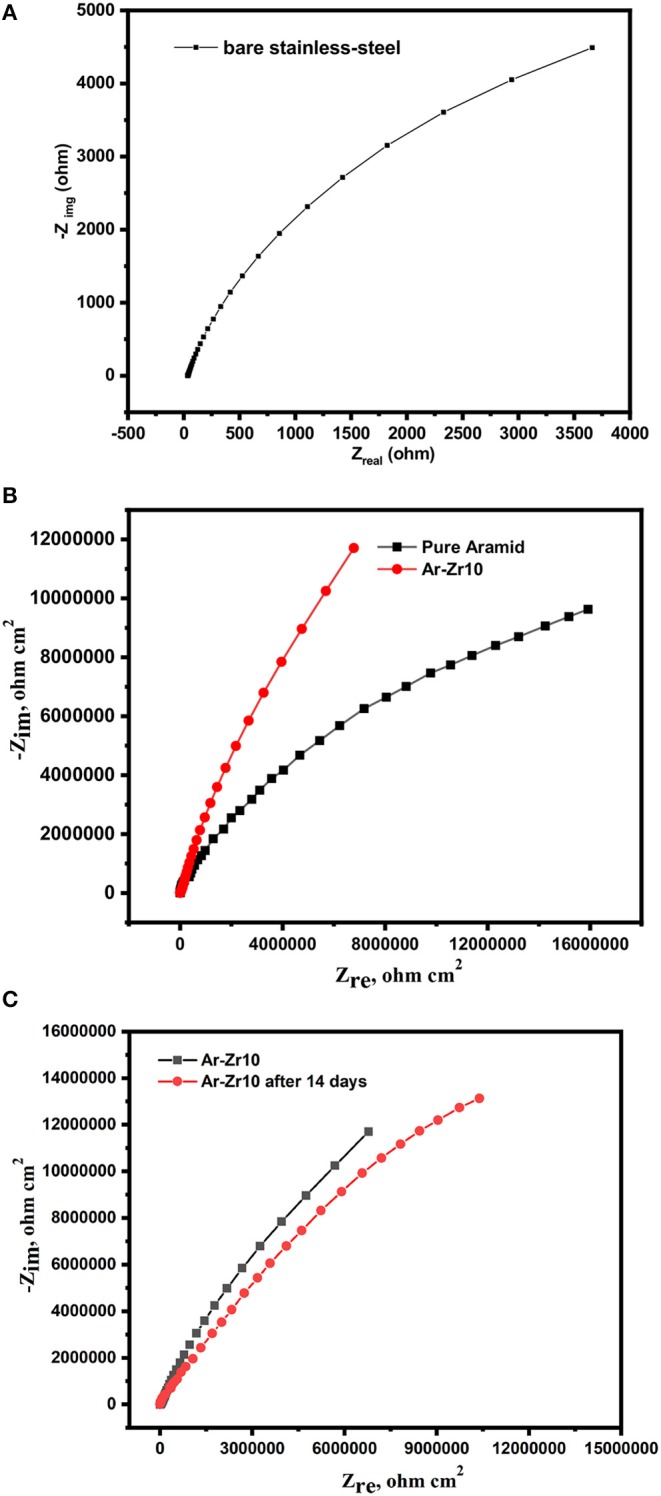
Nyquist plots for bare stainless-steel alloy alone **(A)**, coated with pure aramid and Ar-Zr10 composite **(B)**, and coated with Ar-Zr10 composite after immersion for 14 days **(C)** in 3.5% NaCl solution.

EIS spectra for the uncoated stainless-steel alloy, the alloy coated by pure aramid, and the Ar-Zr10 composite in a solution of 3.5% NaCl are depicted in Figure 6. The Nyquist plot of the bare stainless-steel alloy (Figure 6A) is described by a depressed capacitive loop in the high and medium frequency ranges, due to the charge transfer process. Due to the result obtained from the bare stainless-steel alloy being masked by the coatings curves we separated it into two figures, and this confirms the higher impedance recorded in the case of the coatings under investigation (Figure 6B). While all the curves showed the same semi-circle shape, their sizes differed considerably. The diameter of EIS curves markedly increases in the case of coatings, with the greater diameter recorded using the Ar-Zr10 composite coating compared to the pure aramid coating.

**Figure 7:**
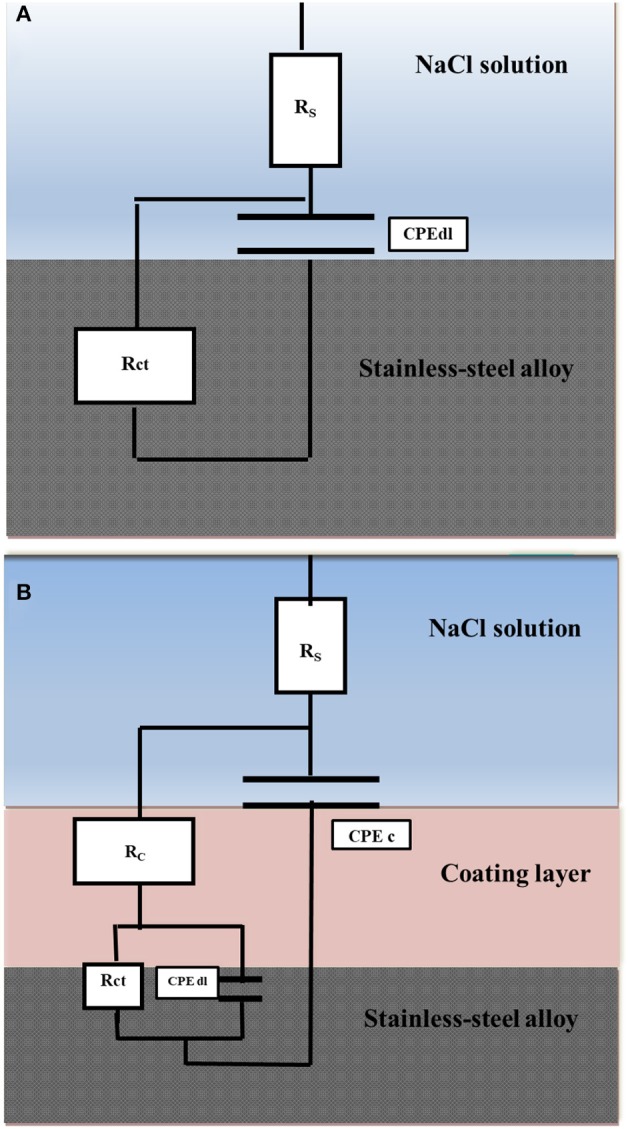
Equivalent circuits used to fit the EIS data of the bare stainless-steel alloy **(A)** and the coated stainless-steel alloy **(B)**.

Figure 7A displays a scheme of the circuit used to match the results attained for the bare stainless-steel alloy. ZSimpWin software was used for the data fitting. The circuit consists of R_s_ (resistance of solution), R_ct_ (resistance of charge transfer), and C_dl_ (capacitance of double layer). The capacitive element was replaced by a constant phase element (CPE) in order to achieve its best fit. In case of the examined coatings, two-time constants were used for fitting the results as depicted in Figure 7B, in which R_c_ and CPE_c_ are ascribed to the coatings.

According to the results, a noticeable increase in R_ct_ when using the investigated coatings was obtained relative to the bare stainless-steel alloy (4.1 × 10^3^ Ωcm^2^). The results confirm the protected surface of the stainless-steel alloy and hence the corrosion rate decrease. The higher R_ct_ value was recorded by the Ar-Zr10 composite (6.7 × 10^5^ Ωcm^2^) compared to (1.12 × 10^5^ Ωcm^2^) in the case of the pure aramid coating. These results prove the better protection against corrosion using the composite coating. Additionally, although the immersion time of the Ar-Zr10 composite coating increased to seven days, there was a slight decrease observed in R_ct_ (4.12 × 10^5^ Ωcm^2^) and the coating showed pronounced protection behavior. With the increase of immersion time of the Ar-Zr10 composite coating to 14 days, a decrease in the R_ct_ was recorded (2.91 × 10^5^ Ωcm^2^) while the composite coating still maintained efficient protection (Figure 6C). Additionally, as the R_ct_ and CPE have a reversible relationship, the CPE_dl_ values obtained in the presence of the investigated coating showed a marked decrease with the lowest CPE_dl_ value using of the aramid-zirconia (10%) composite (7.3 × 10^−7^ Ω^−1^ cm^−2^ s^n^) in comparison with the bare stainless-steel alloy of (2.3 × 10^−5^ Ω^−1^ cm^−2^ s^n^). Decreasing the CPE_dl_ values in the presence of the examined coatings was associated with an increase in the thickness of the electrical double layer and/or the decrease of the dielectric constant, that proved the great adhesion of the coatings on the stainless-steel alloy surface (Nazeer et al., 2012). Generally, the EIS results in the high frequency region are related to both the compactness and the hydrophobic characteristics of the surface, while the EIS modulus at a low frequency is associated with the corrosion resistance behavior (Chang et al., 2014).

The improvement in the presence of the Ar-Zr10 composite coating suggests blocking the ionic conducting pathways might present as pores or defects in the aramid coating with zirconia particles. Also, the polarization and impedance findings proved that the Ar-Zr10 composite coating can effectively suppress or delay penetrating the corrosive anions of NaCl via the coating and improve the corrosion barrier characteristic.

### Wettability Test of the Stainless-Steel Alloy Coated Surfaces

**Figure 8:**
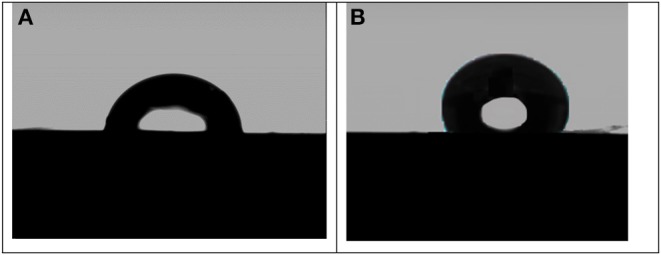
Contact angles of stainless steel electrodes coated with pure aramid **(A)** and with aramid-zirconia (10%) composite **(B)**.

The contact angle (CA) test is of one of the most practical tests of surface modification using a sessile drop process. Figure 8 shows the water contact angles images of the stainless steel coated with the pure aramid and coated with aramid-zirconia (10%) composite measured at room temperature. From this figure, it was observed that the contact angle in the presence of the pure aramid coating exhibited a water contact angle of 84.2°. Adding the zirconia to the aramid polymer has led to significant improvement in the wettability with a water CA of 125°, and this could be attributed to the rough surface of the zirconia in the composite film, which improves the hydrophobicity (Rezaei et al., 2018). From the results, the surface hydrophobicity of the stainless steel surface was considerably improved in the presence of the investigated hybrid coating (Ar-Zr10) compared to the pure aramid coating.

Additionally, the hydrophobic Ar-Zr10 composite film has the great ability to prevent the absorption of corrosive ions and water molecules. In general, the improvement of the hydrophobic property will enhance the metal substrate protection, eliminating the corrosion attack. Consequently, the corrosive ions and water molecules will take a long time for diffusion from the solution interface to the surface of the coating. Also, the presence of zirconia increases the composite coating roughness and the water CA which suppress the corrosion process.

### Microstructure and Composition of the Examined Coatings

**Figure 9:**
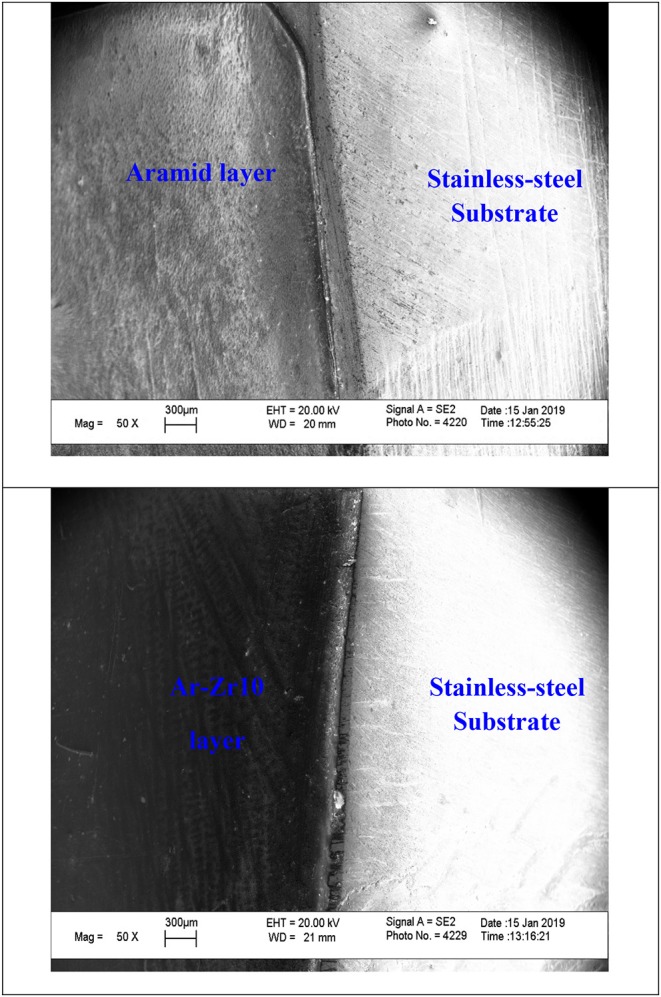
SEM images of the cross-sectional morphology of the stainless steel alloy surfaces coated with pure aramid and Ar-Zr10 composite coating.

Figure 9 displays the cross sectional morphology of the pure aramid and the Ar-Zr10 composite coatings on a stainless steel alloy. The image of the coatings on the steel alloy shows complete surface coverage of the coatings to the bare steel. The uniform layer formed on the stainless steel alloy using the Ar-Zr10 composite proves the lack of zirconia aggregation and its good dispersion in the coating film. In addition, the coatings thicknesses are found to be in the range of about 40-50 μm.

**Figure 10:**
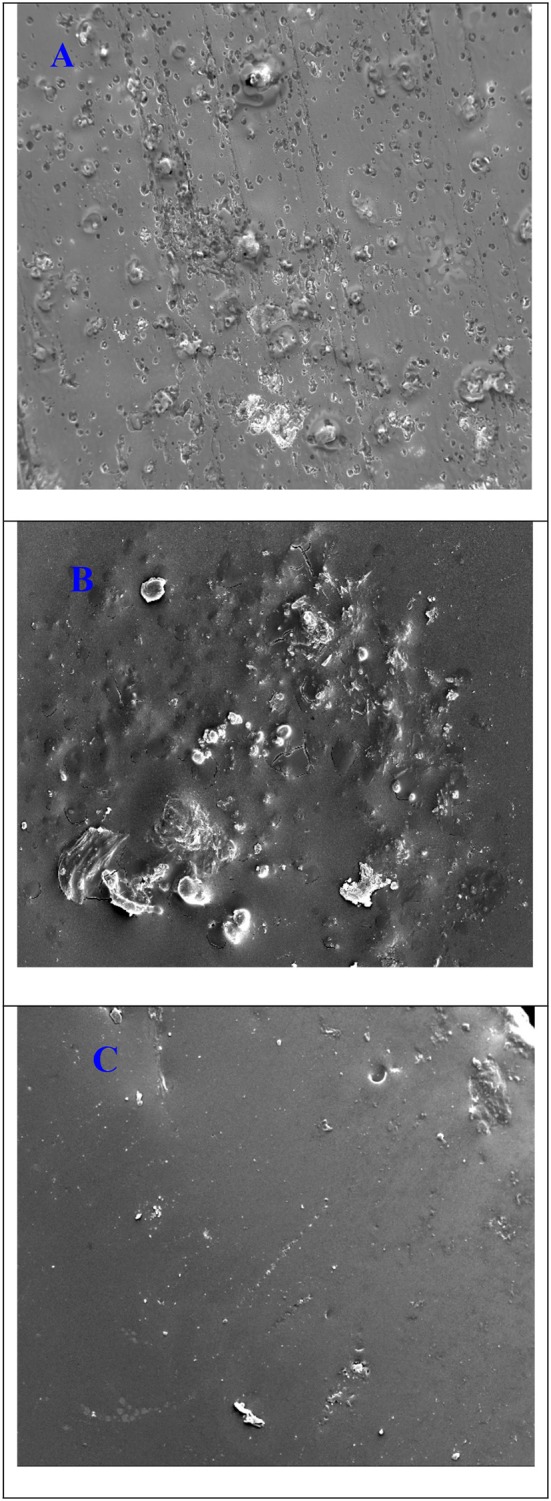
SEM micrographs of **(A)** the uncoated stainless-steel alloy, **(B)** the pure aramid coating, and **(C)** the Ar-Zr10 hybrid coating immersed in 3.5% NaCl solution for 14 days.

Figure 10 depicts the surface morphology of the coated and uncoated stainless steel alloy with pure aramid and Ar-Zr10 composite coating after immersion in 3.5% NaCl solution for 14 days. In the bare stainless-steel alloy (Figure 10A), it is obvious that the surface was damaged and corroded with the existence of various cracks and pits. In the case of the pure aramid, there were some cracks with corrosion products and very few pits (compared with the bare stainless steel) (Figure 10B). While in the case of the Ar-Zr10 coating (Figure 10C), a small amount of corrosion products has been observed, confirming its greater resistance compared to the pure aramid. The presence of the hybrid coating showed a smoother surface with better protection relative to the pure aramid coating, which is in line with the data collected from the various examined techniques.

### Anticipated Protection Mechanism

The corrosion process of the coated metals and alloys surfaces mainly occurs due to the water adsorption on the surface accompanied by a penetrating of the coating with corrosive ions of the corrosive electrolyte reaching the interface of the substrate/coating (Wei et al., 2013). The corrosive ions of the electrolyte are able to initiate the corrosion process, which includes different redox reactions. Therefore, suitable coatings are required to overcome the corrosive ions diffusion to the metallic surface and suppress the possible redox reactions.

Consequently, the investigated hydrophobic composite polymer diminished the water adsorption on the steel surface and resulted in considerable protection from the corrosion process. This protection was markedly improved when using the Ar-Zr10 composite coating. The pronounced protection in the presence of the composite coating is believed to be attributed to its prevention of the aggressive ions from penetrating the coating by acting as an effective barrier. Also, the presence of zirconia could improve the adhesion of the composite coating on the steel surface by forming a strong bond (Zr-O-metal). Moreover, the well-dispersed zirconia in the aramid polymer led to a marked decrease in the coating surface energy and increased the CA to 125° which improves its hydrophobicity and resulted in excellent corrosion protection.

## Conclusion

In conclusion, an environmentally benign sol-gel method was used to prepare the aramid polymer and an organic-inorganic hybrid film of the Ar-Zr10 composite. The investigated films were fully characterized to confirm their successful preparation. The thermal degradation of the hybrid film in air using the TGA technique confirms the respected percent of zirconia in the composite with 10%. The prepared films were applied as coatings for stainless-steel corrosion protection. The Ar-Zr10 composite demonstrated greater corrosion protection behavior compared to the pure aramid and the bare stainless-steel. Embedding zirconia in the aramid coating enhanced the barrier characteristics of the pure aramid coating. PDP and EIS results exhibited pronounced protection efficiency in the presence of the Ar-Zr10 nanocomposite. Marked protection was recorded after immersing the coated steel alloy with the composite coating for 14 days in the chloride medium. According to the electrochemical results, the Ar-Zr10 nanocomposite coating acted as a physical barrier by suppressing the water molecules and the aggressive ions diffusion to the steel surface, which enhanced the steel resistance and prevented the corrosion process. The results showed that the tested coatings can be considered suitable for stainless steel protection in different applications and can be applied to different metals and alloys.

## Data Availability Statement

All datasets generated for this study are included in the article/supplementary material.

## Author Contributions

AB was the principal investigator of the research in this work and proposed the idea, supervised the experimental work, analyzed the results, and helped in writing the manuscript. FA was the co-investigator of the research in this work, helped in proposing the idea of the research and supervised the experimental work, analyzed the results, and helped in writing the manuscript. AN designed the corrosion study, conducted the other experimental work, and drafted the manuscript.

## Conflict of Interest

The authors declare that the research was conducted in the absence of any commercial or financial relationships that could be construed as a potential conflict of interest.
